# Estrogen Receptors Promote Migration, Invasion and Colony Formation of the Androgen-Independent Prostate Cancer Cells PC-3 Through β-Catenin Pathway

**DOI:** 10.3389/fendo.2020.00184

**Published:** 2020-04-09

**Authors:** Ana Paola G. Lombardi, Carolina M. Vicente, Catarina S. Porto

**Affiliations:** Laboratório de Endocrinologia Experimental, Departamento de Farmacologia, Escola Paulista de Medicina, Universidade Federal de São Paulo, São Paulo, Brazil

**Keywords:** ERβ, β-catenin, VEGFA, PC-3 cells, ERα

## Abstract

Prostate cancer is initially dependent on the androgen, gradually evolves into an androgen-independent form of the disease, also known as castration-resistant prostate cancer (CRPC). At this stage, current therapies scantily improve survival of the patient. Androgens and estrogens are involved in normal prostate and prostate cancer development. The mechanisms by which estrogens/estrogen receptors (ERs) induce prostate cancer and promote prostate cancer progression have not yet been fully identified. Our laboratory has shown that androgen-independent prostate cancer cells PC-3 express both ERα and ERβ. The activation of ERβ increases the expression of β-catenin and proliferation of PC-3 cells. We now report that the activation of ERβ promotes the increase of migration, invasion and anchorage-independent growth of PC-3 cells. Furthermore, the activation of ERα also plays a role in invasion and anchorage-independent growth of PC-3 cells. These effects are blocked by pretreatment with PKF 118–310, compound that disrupts the complex β-catenin/TCF/LEF, suggesting that ERs/β-catenin are involved in all cellular characteristics of tumor development *in vitro*. Furthermore, PKF 118–310 also inhibited the upregulation of vascular endothelial growth factor A (VEGFA) induced by activation of ERs. VEGF also is involved on invasion of PC-3 cells. In conclusion, this study provides novel insights into the signatures and molecular mechanisms of ERβ in androgen-independent prostate cancer cells PC-3. ERα also plays a role on invasion and colony formation of PC-3 cells.

## Introduction

Prostate cancer is the prevalent cancer diagnosed among men. A better understanding of the expression of androgen and estrogen receptors associated with disease initiation and progression is critical for better diagnosis and treatment (1). Prostate cancer is initially dependent on the androgen, gradually evolves into an androgen-independent form of the disease, also known as castration-resistant prostate cancer (CRPC) [(1); reviewed by (2–5)]. At this stage, current therapies scantily improve survival of the patient.

Androgens and estrogens are involved in normal prostate and prostate cancer development [reviewed by (5–8)]. It has not yet been fully identified, the mechanisms by which estrogens induce prostate cancer and promote prostate cancer progression [reviewed by (4, 5, 9, 10)].

The expression of estrogen receptors ERα and ERβ changes in different stages of the prostate cancer and conflicting findings on the roles of these receptors in the prostate cancer continue to emerge [reviewed by (11–15)]. However, studies have shown that ERβ may be potentially oncogenic in prostate cancer (16–19).

In androgen-independent prostate cancer cells PC-3 (20) and DU-145 (21), ERα and ERβ are mostly located in the extranuclear region of these cells. The activation of these receptors increases the phosphorylation of ERK1/2 (extracellular signal-regulated kinase1 and 2) and AKT (serine/threonine kinases) in PC-3 cells (18, 20). On the other hand, phosphorylated AKT is not detected in DU-145 cells (21). The role of these pathways through activation of estrogen receptors (ERs) remains to be explored in both cells. Furthermore, in PC-3 cells, the activation of ERβ decreases N-cadherin (22) and increases non-phosphorylated β-catenin levels (18). However, the molecular mechanisms of the crosstalk between ER and WNT/β-catenin pathways in this cell have not been well studied.

Activation of TCF/LEF (T-cell-specific transcription factor/lymphoid enhancer-binding factor) by β-catenin induces growth, migration, and invasion of prostate cancer cells (23–25) and maintains the stemness of the cancer stem cells (26). Moreover, chemotherapy resistance is observed (27).

In this study, we aimed to examine the role of estrogen receptors and β-catenin on migration, invasion and colony formation of the androgen-independent prostate cancer cells PC-3.

## Materials and Methods

### Cell Culture

The human androgen-independent prostate cancer cell line (PC-3) (derived from bone metastasis) was obtained from the American Type Culture Collection (Manassas, VA, United States). PC-3 cells cultures were carried out as previously described (18, 20–22).

All experimental procedures were approved by the Research Ethical Committee at Escola Paulista de Medicina-Universidade Federal de São Paulo (#4330100615).

### Wound Healing Analysis

For wound scratch assay, 2 × 10^5^ cells were grown in a 24-well plate. PC-3 cells were washed with PBS and incubated in serum free culture medium containing a blocking DNA replication mitomycin C (10 μg/L) to avoid cell proliferation. The cells were wounded using 200 μl sterile pipette tips and then washed to remove detached cells and debris (28) and incubated in the absence (control, basal level of cellular function) and presence of 17β-estradiol (E2, 0.1 and 10 nM; Sigma Chemical Co.); ERβ-selective agonist DPN [10 nM; 2,3-bis(4-hydroxyphenyl)-propionitrile, Tocris Bioscience, Bristol, United Kingdom] and ERB-041 (10 nM; Sigma Chemical Co.) or ERα-selective agonist PPT [10 nM; 4,4’,4”-(4-propyl-(1H)-pyrazole-1,3,5-triyl)trisphenol, Tocris Bioscience] for 24 h at 37°C. The cells were also untreated or pretreated with ERβ-selective antagonist PHTPP [10 nM; 4-(2-phenyl-5,7-bis(trifluoromethyl)pyrazolo(1,5-a)pyrimidin-3-yl)phenol, Tocris Bioscience] or with a compound that disrupts the complex β-catenin-TCF/LEF transcription factor, PKF 118–310 (100 nM, Sigma-Aldrich Co) for 30 min. Incubation was continued in the absence and presence of DPN (10 nM) or ERB-041 (10 nM), for 24 h at 37°C (18, 21, 22). The agonists and antagonists are highly selective, at these concentrations, as previously reported [(29, 30), (31), (20)]. The concentration of PKF 118–310 used did not change the PC-3 cell viability (32), but antagonize cellular effects of β-catenin-dependent activities in PC-3 cells (18) and in soft tissue sarcoma (STS) cells (33). For measuring the closure of the wound in control (untreated cells) and treated cells, photographs of the same area of the wound were taken at 0 and 24 h (34). Images were captured using a Nikon Eclipse inverted optical microscope and analyzed by Micrometrics SE Premium 4 software. The areas that were occupied by migrating cells after 24 h of incubation (control and treated cells) were calculated by subtracting the background levels at 0 h. Results were plotted (mean ± SEM) in relation to control (*C* = 1) or in relation to agonists subtracted from the control (agonists = 1). Images are representative of three to six different experiments.

### Cell Invasion Analysis

PC-3 cells (2 × 10^5^ cells) in serum free culture medium were seeded in Thincert^®^ chambers (Greiner Bio-one, Kremsmünster, Austria) with polyethylene terephthalate membranes (8 μm pore size) (28) pre-coated with 50 μl of phenol red-free Matrigel (1:10, BD, Corning). These chambers were placed in 24-well plates containing culture medium with 10% FBS in the lower chamber. PC-3 cells in upper chambers were incubated in the absence (control) and presence of E2 (10 nM), DPN (10 nM) or PPT (10 nM) for 48 h at 37°C. The cells were also untreated or pretreated with PHTPP (10 nM), MPP [10 nM; 1,3-bis(4-hydroxyphenyl)-4-methyl-5-(4-(2-piperidinylethoxy)phenol)-1H-pyrazole dihydrochloride, Tocris Bioscience], PKF 118–310 (100 nM) (18, 21, 22) or VEGF specific inhibitor Bevacizumab (Avastin^®^, 25 ng) (35) for 30 min. Incubation was continued in the absence and presence of E2 (10 nM), DPN (10 nM) or PPT (10 nM), for 48 h at 37°C. The membranes were washed thoroughly with 10 mM PBS, fixed in 4% paraformaldehyde for 30 min, and stained with 0.2% crystal violet for 10 min (28). Non-invading cells from the membrane upper surface were removed using a sterile cotton swab. The membranes containing the invaded cells (under the surface of membrane), were photographed. Images of three random microscope fields, in duplicate, were captured using an inverted optical microscope (Floid Cell Imaging Station, Life Technologies). The areas of invaded cells were determined by Image J software. Results were plotted (mean ± SEM) in relation to control (*C* = 100) or in relation to agonists subtracted from the control (agonists = 100). Images are representative of three different experiments.

### Colony Formation Analysis (Soft Agar)

PC-3 cells (6 × 10^3^ cells) in culture medium containing 10% FBS and 0.35% agarose (low melting 0.7%, Sigma Chemical Co.) were seeded in 24-well plates pre-coated with 300 μl of 0.7% agarose at 4°C for 30 min (28). Cells were incubated at 37°C for 2 h. Afterward, this culture medium was replaced by culture medium containing 10% SFB, pretreated with activated charcoal (0.25%) and dextran T-70 (0.0025%), for 24 h at 37°C. PC-3 cells were incubated in the absence (control) and presence of E2 (10 nM), DPN (10 nM) or PPT (10 nM) for 3 weeks, with regular change in medium on every alternate day, at 37°C. Cells were also untreated or pretreated with PHTPP (10 nM), MPP (10 nM) or PKF 118–310 (100 nM) for 30 min. Incubation was continued in the absence and presence of DPN (10 nM) or PPT (10 nM), for 3 weeks at 37°C (18, 21, 22). Images of three random microscope fields, in duplicate, were captured using an inverted optical microscope (Floid Cell Imaging Station, Life Technologies). The area of each colony was determined by Image J software; only the spheroid-shaped colonies were considered for the area calculation. Images of four random microscope fields, in duplicate, were also captured using an inverted optical microscope (AxioObserverZ1) for determination of the number of colonies. Star- and spheroid-shaped colonies above 50 μm were counted using software Zen. Images are representative of three different experiments.

### Immunofluorescence Analysis for the Detection of VEGF

PC-3 cells were grown as described above on coverslips coated with gelatin (0.1%, w/v) and placed into six-well plates. PC-3 cells in serum free culture medium were incubated in the absence (control) and presence of E2 (10 nM); DPN (10 nM) or PPT (10 nM), for 48 h at 37°C. The cells were also untreated or pretreated with PKF 118–310 (100 nM) for 30 min. Incubation was continued in the absence and presence of E2 (10 nM), PPT (10 nM) or DPN (10 nM), for 48 h at 37°C (18, 21, 22). The immunofluorescence analyses were performed as previously described [(20), (18)], using a rabbit polyclonal antibody raised against a peptide 1-140 of VEGFA of human origin (sc-507, Santa Cruz Biotecnology Inc., Dallas, TX, United States) at 1:100 dilution and Alexa Fluor 488-labeled secondary antibody (anti-rabbit antibody at 1:300 dilution; Molecular Probes^®^, Invitrogen, NY, United States). Images of five random microscope fields were captured, in duplicate, in each assay (two different experiments) and analyzed using the software LAS-AF and ImageJ. Images are representative of at least two different experiments performed in duplicate.

### Statistical Analysis

Data were expressed as mean ± SEM. Statistical analysis was carried out by Student *t*-test to compare the differences between two data. *P* values < 0.05 were accepted as significant.

## Results

### Activation of ERβ, but Not ERα, Increases PC-3 Cells Migration

**FIGURE 1:**
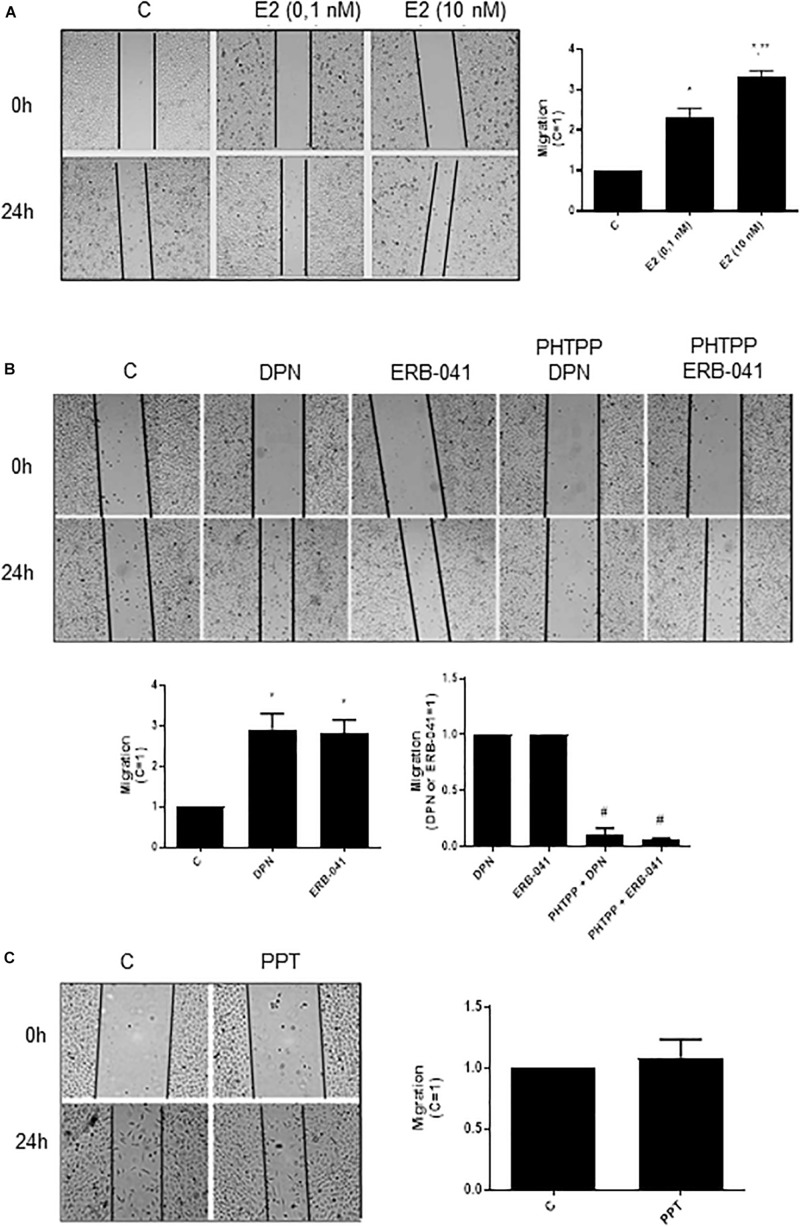
Effects of 17β-estradiol (E2), ERβ-selective agonists DPN or ERB-041 and ERα-selective agonist PPT on PC-3 cells migration. Cells were incubated in culture medium containing mitomycin C (10 μg/L) to avoid cell proliferation. Cells were wounded and then incubated in the absence (C, control) and presence of E2 (0.1 and 10 nM) **(A)**, DPN (10 nM), ERB-041 (10 nM) **(B)**, or PPT (10 nM) **(C)** for 24 h at 37°C. Cells were also untreated or pretreated with the ERβ-selective antagonist PHTPP (10 nM) for 30 min. Incubation was continued in the absence and presence of DPN or ERB-041 for 24 h at 37°C **(B)**. Photographs of the same area of the wound were taken at 0 and 24 h. Images were captured using an inverted optical microscope and analyzed by Micrometrics SE Premium 4 Software. The areas that were occupied by migrating cells after 24 h of incubation (control and treated cells) were calculated by subtracting the background levels at 0 h. Results were plotted (mean ± SEM) in relation to control (*C* = 1) or in relation to agonists subtracted from the control (agonists = 1). *Significantly different from control, C (*P* < 0.05, Student *t*-test). **Significantly different from E2 (0.1 nM) (*P* < 0.05, Student *t*-test). #Significantly different from DPN or ERB-041 (*P* < 0.05, Student *t*-test). No statistical difference was observed with PPT (*P* > 0.05, Student *t*-test). Images are representative of four different experiments.

In PC-3 cells, previous study from our laboratory showed that 17β-estradiol regulates the cell proliferation (18). In the present study, we analyzed different cellular characteristics of tumor development *in vitro*. Cell migration (cell motility) was evaluated by the wound scratch analysis in PC-3 cells. For measuring the closure of the wound in control (C) (basal level of cellular function) and treated cells, photographs of the same area of the wound were taken at 0 and 24 h. At 24 h treatment, 17β-estradiol (E2 0.1 and 10 nM) increased the migration of PC-3 cells (two- and three-fold) (Figure 1A) compared to control (C) (Figure 1A).

Similar results were observed with ERβ-selective agonists DPN (10 nM) and ERB-041 (10 nM) (three-fold compared to control) (Figure 1B). These effects were blocked by pretreatment of the cells with ERβ-selective antagonist PHTPP (Figure 1B). On the other hand, the ERα-selective agonist PPT (10 nM) did not modify the migratory capacity of PC-3 cells (Figure 1C). The treatment with ERβ-selective antagonist PHTPP alone did not have any effect on basal level of cellular function (Supplementary Figure S1). All together, these results indicate the involvement ERβ on migration of PC-3 cells.

### Activation of ERβ and ERα Increases PC-3 Cells Invasion

**FIGURE 2:**
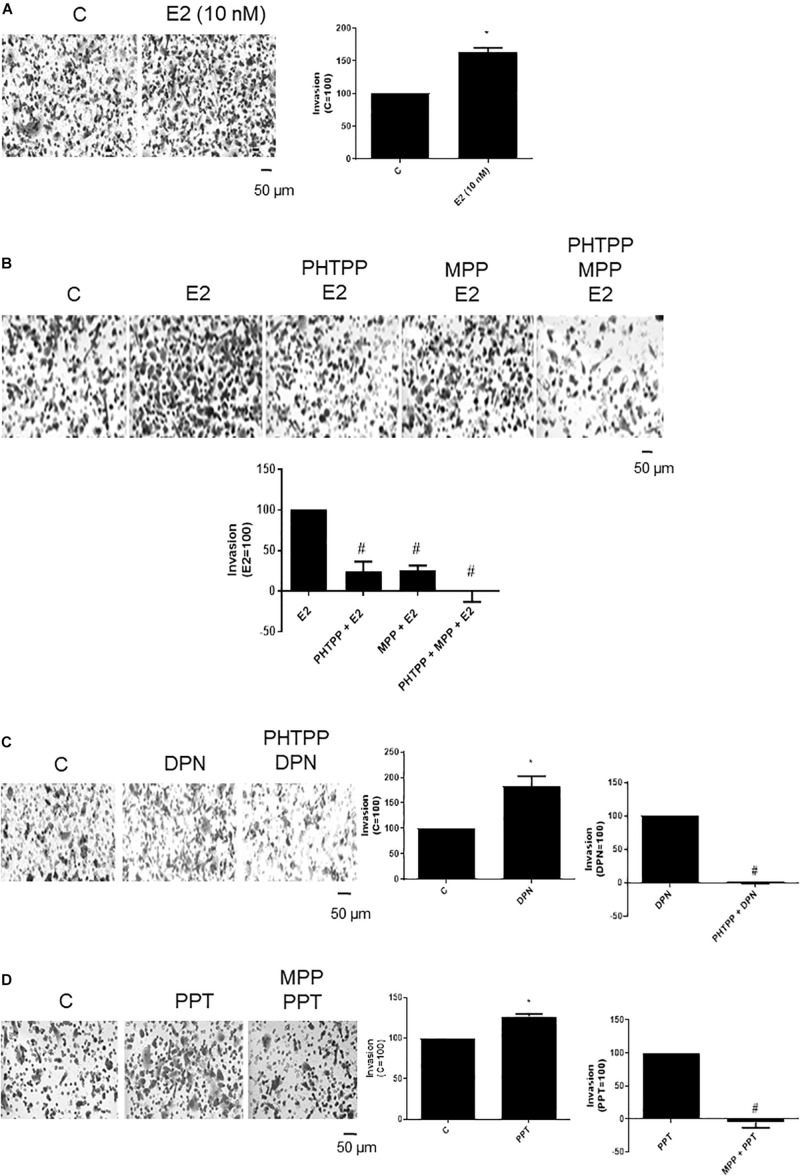
Effects of 17β-estradiol (E2), ERβ-selective agonist DPN and ERα-selective agonist PPT on PC-3 cells invasion. Cells in serum free culture medium were seeded in Thincert^®^ chambers with polyethylene terephthalate membranes pre-coated with phenol red-free Matrigel. These chambers were placed in 24-well plates containing culture medium with 10% FBS in the lower chamber. Cells in upper chambers were incubated in the absence (C, control) and presence of 17β-estradiol (E2, 10 nM) **(A,B)**, ERβ-selective agonist DPN (10 nM) **(C)** or ERα-selective agonist PPT (10 nM) **(D)** for 48 h at 37°C. Cells were also untreated or pretreated with ERβ-selective antagonist PHTPP (10 nM), with ERα-selective antagonist MPP (10 nM), or with both antagonists, PHTTP (10 nM) and MPP (10 nM) for 30 min. Incubation was continued in the absence and presence of E2, DPN, or PPT for 48 h at 37°C **(B–D)**. The membranes containing the invaded cells (under the surface of membrane), were photographed. Images of three random microscope fields, in duplicate, were captured using an inverted optical microscope. The areas of invaded cells were determined by Image J software. Results were plotted (mean ± SEM) in relation to control (*C* = 100) or in relation to agonists subtracted from the control (agonists = 100). *Significantly different from control, C (*P* < 0.05, Student *t*-test). #Significantly different from E2, DPN or PPT (*P* < 0.05, Student *t*-test). Scale bar as indicated. Images are representative of three different experiments.

To examine the influence of ERβ and ERα on the invasiveness of prostate cancer cells, Matrigel transwell invasion assays were carried out. Treatment with E2 (10 nM, 48 h) caused an enhancement on invasion of PC-3 cells (60%) (Figure 2A), suggesting the involvement of estrogen receptors in this process. In fact, the increase of PC-3 cells invasion induced by E2 was partially blocked by ERβ- (PHTTP) (76%) and ERα- (MPP) (75%) selective antagonists (Figure 2B). Simultaneous pretreatment with both PHTTP and MPP completely blocked the effect of E2 (100%) (Figure 2B), suggesting that both receptors ERβ and ERα may play a role in the PC-3 cells invasion.

DPN (10 nM) or PPT (10 nM) also increased the PC-3 cell invasion (80% and 27%, respectively) (Figures 2C,D). These effects were blocked by pretreatment with respective ERβ- and ERα-selective antagonists (Figures 2C,D), confirming the involvement of both receptors on cell invasion.

The treatment with ERα (MPP)- or ERβ (PHTTP)-selective antagonists alone did not have any effects on basal level of cellular function (Supplementary Figure S2).

### Activation of ERβ and ERα Increases the Size and Number of the Colony Formed by PC-3 Cells

**FIGURE 3:**
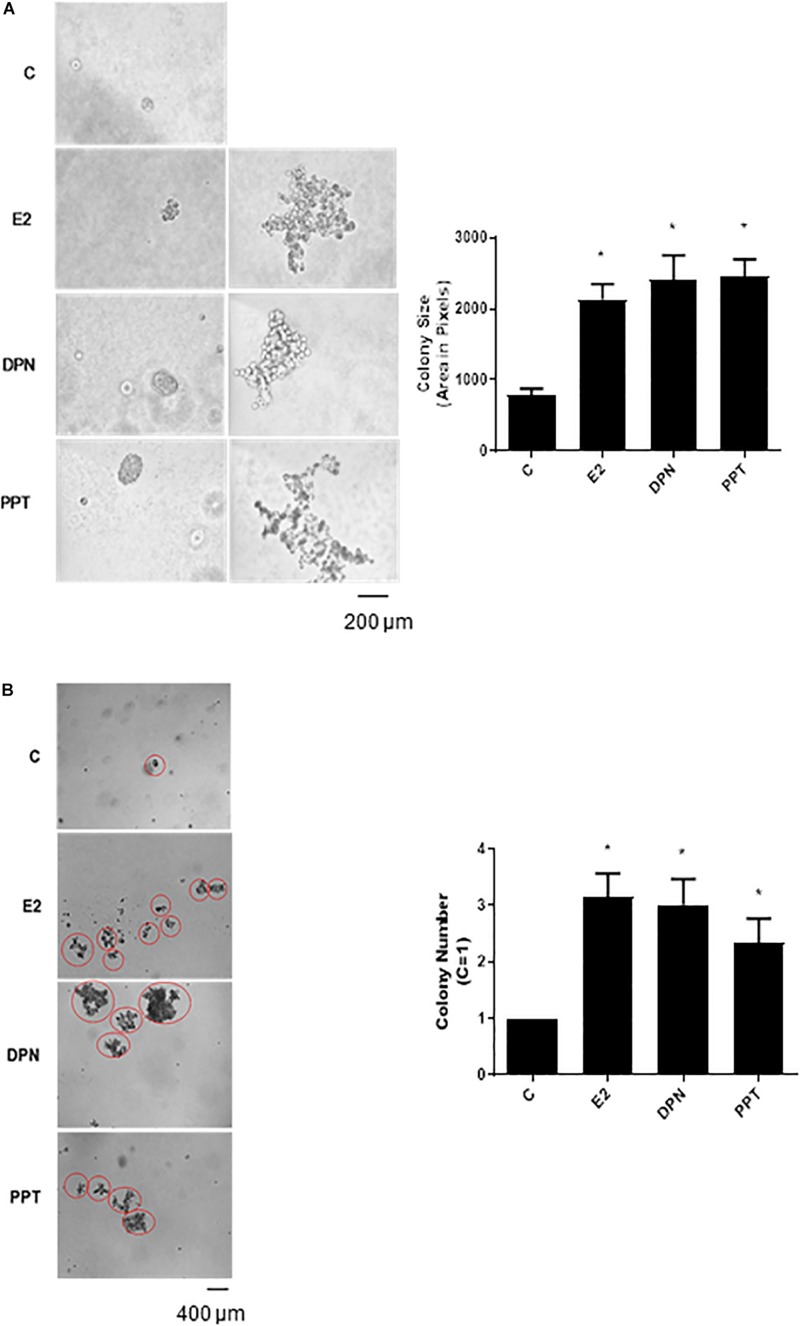
Effects of 17β-estradiol (E2), ERβ-selective agonist DPN, and ERα-selective agonist PPT on size and number of colonies formed by PC-3 cells. PC-3 cells in culture medium containing 10% FBS and 0.35% agarose (low melting 0.7%) were seeded in 24-well plates pre-coated 0.7% agarose. Cells were incubated for 2 h at 37°C. Afterward, culture medium containing 10% SFB pretreated with activated charcoal (0.25%) and dextran T-70 (0.0025%) was added for 24 h at 37°C. After that, cells were incubated in the absence (C, control) and presence of 17β-estradiol (E2, 10 nM), ERβ-selective agonist DPN (10 nM) or ERα-selective agonist PPT (10 nM) for 3 weeks, with regular change in medium on every alternate day, at 37°C. Images of three random microscope fields, in duplicate, were captured using an inverted optical microscope. The area of each colony was determined by Image J Software; only the spheroid-shaped colonies were considered for the area calculation **(A)**. Images of four random microscope fields, in duplicate, were also captured using an inverted optical microscope for determination of the number of colony **(B)**. Star- and spheroid-shaped colonies above 50 μm were counted using software Zen. *Significantly different from control, C (*P* < 0.05, Student *t*-test). Scale bar as indicated. Images are representative of three different experiments.

To further analyze the tumorigenic potential of estrogen receptors in PC-3 cells, we performed the colony formation, anchorage-independent growth assay. E2 (10 nM), DPN (10 nM), and PPT (10 nM) at third weeks of treatment increased the size and number of the colony formed by PC-3 cells (round spheroids structures) compared to control (C) (Figures 3A,B).

Furthermore, E2, DPN, and PPT also induced PC-3 stellate or invasive phenotype (Figure 3A). The increase in round PC-3 spheroids (size and number) induced by DPN and PPT was blocked by ERβ- (PHTTP) and ERα- (MPP) selective antagonists, respectively (data not shown), indicating that both receptors may play a role in the size and number of the colony formation.

### β-Catenin Is Involved in Migration, Invasion, and Colony Formation of PC-3 Cells

**FIGURE 4:**
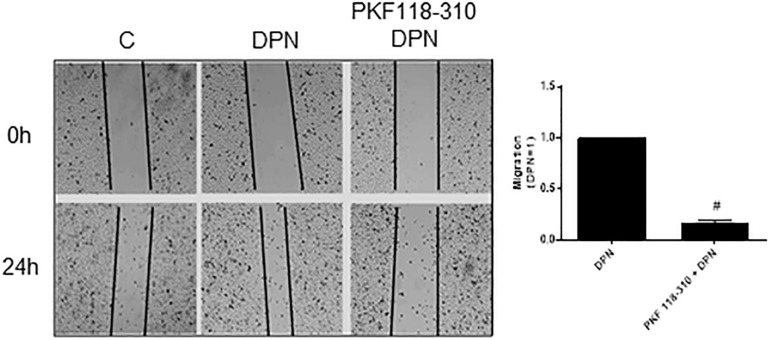
Effects of the compound that disrupts the complex β-catenin-TCF/LEF PKF 118–310 on DPN-induced PC-3 cells migration. Cells were incubated in culture medium containing mitomycin C (10 μg/L) to avoid cell proliferation. Cells were wounded and then incubated in the absence (C, control) and presence of DPN (10 nM), for 24 h at 37°C. Cells were also untreated or pretreated with a compound that disrupts the complex β-catenin-TCF/LEF transcription factor, PKF 118–310 (100 nM) for 30 min. Incubation was continued in the absence and presence of DPN for 24 h at 37°C. Photographs of the same area of the wound were taken at 0 and 24 h. Images were captured using an inverted optical microscope and analyzed by Micrometrics SE Premium 4 Software. The areas that were occupied by migrating cells after 24 h of incubation (control and treated cells) were calculated by subtracting the background levels at 0 h. Results were plotted (mean ± SEM) in relation to DPN subtracted from the control. #Significantly different from DPN (*P* < 0.05, Student *t*-test). Images are representative of three different experiments.

Previous study from our laboratory demonstrated that activation of ERβ by DPN induced an increase in non-phosphorylated β-catenin expression (18). To explore a possible involvement of β-catenin in DPN-induced PC-3 cells migration, we used PKF 118–310. The pretreatment with PKF 118–310 inhibited by 84% the migration of PC-3 cells induced by DPN, indicating the involvement of ERβ/β-catenin in the regulation of PC-3 cells migration (Figure 4).

**FIGURE 5:**
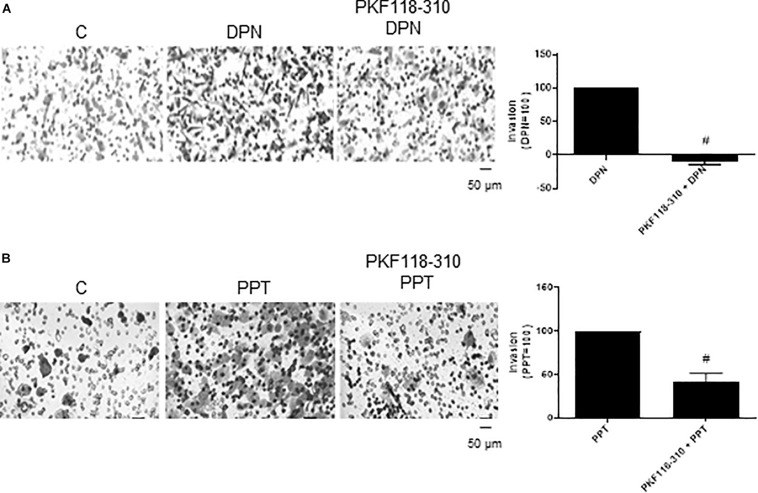
Effects of the compound that disrupts the complex β-catenin-TCF/LEF PKF 118–310 on DPN or PPT-induced PC-3 cells invasion. Cells in serum free culture medium were seeded in Thincert^®^ chambers with polyethylene terephthalate membranes pre-coated with phenol red-free Matrigel. These chambers were placed in 24-well plates containing culture medium with 10% FBS in the lower chamber. Cells in upper chambers were incubated in the absence (C, control) and presence of ERβ-selective agonist DPN (10 nM) **(A)** or ERα-selective agonist PPT (10 nM) **(B)** for 48 h at 37°C. Cells were also untreated or pretreated with a compound that disrupts the complex β-catenin-TCF/LEF transcription factor, PKF 118–310 (100 nM) for 30 min. Incubation was continued in the absence and presence of DPN or PPT for 48 h at 37°C **(A,B)**. The membranes containing the invaded cells (under the surface of membrane), were photographed. Images of three random microscope fields, in duplicate, were captured using an inverted optical microscope. The areas of invaded cells were determined by Image J software. Results were plotted (mean ± SEM) in relation to agonists subtracted from the control (agonists = 100). #Significantly different from DPN or PPT (*P* < 0.05, Student *t*-test). Scale bar as indicated. Images are representative of three different experiments.

In addition, the pretreatment with PKF 118–310 blunted (100%) the effect induced by DPN and reduced (59%) the effect induced by PPT on invasion of PC-3 cells (Figures 5A,B), suggesting also the involvement of ERs/β-catenin on invasion of PC-3 cells.

The treatment with PKF 118–310 alone did not have any effect on migration or invasion of PC-3 cells (Supplementary Figures S1, S2).

**FIGURE 6:**
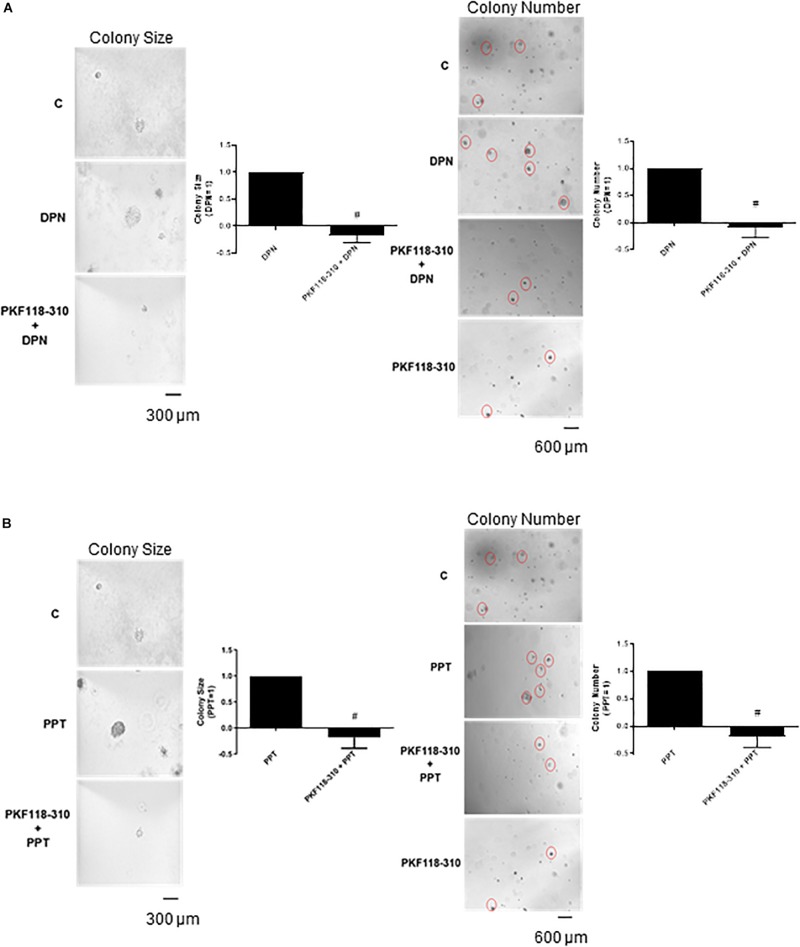
Effects of the compound that disrupts the complex β-catenin-TCF/LEF PKF 118–310 on DPN or PPT-induced colony formation of PC-3 cells. PC-3 cells in culture medium containing 10% FBS and 0.35% agarose (low melting 0.7%) were seeded in 24-well plates pre-coated 0.7% agarose. Cells were incubated for 2 h at 37°C. Afterward, culture medium containing 10% SFB pretreated with activated charcoal (0.25%) and dextran T-70 (0.0025%) was added for 24 h at 37°C. After that, cells were incubated in the absence (C, control) and presence of ERβ-selective agonist DPN (10 nM) **(A)** or ERα-selective agonist PPT (10 nM) **(B)** for 3 weeks, with regular change in medium on every alternate day, at 37°C. Cells were also untreated or pretreated with a compound that disrupts the complex β-catenin-TCF/LEF transcription factor PKF 118–310 (100 nM) for 30 min. Incubation was continued in the absence and presence of DPN or PPT, for 3 weeks at 37°C **(A,B)**. Images of three random microscope fields, in duplicate, were captured using an inverted optical microscope. The area of each colony was determined by Image J Software; only the spheroid-shaped colonies were considered for the area calculation. Images of four random microscope fields, in duplicate, were also captured using an inverted optical microscope for determination of the number of colonies. Star- and spheroid-shaped colonies above 50 μm were counted using software Zen. #Significantly different from DPN or PPT (*P* < 0.05, Student *t*-test). Scale bar as indicated. Images are representative of three different experiments (the control and all treatments were performed in the same experiment; thus, the same control was used for **A,B**).

Finally, the pretreatment with PKF 118–310 blunted the effect induced by DPN and PPT on size and number of the colony formed by PC-3 cells (Figures 6A,B), indicating the involvement of ERs/β-catenin in tumor formation *in vitro*.

**FIGURE 7:**
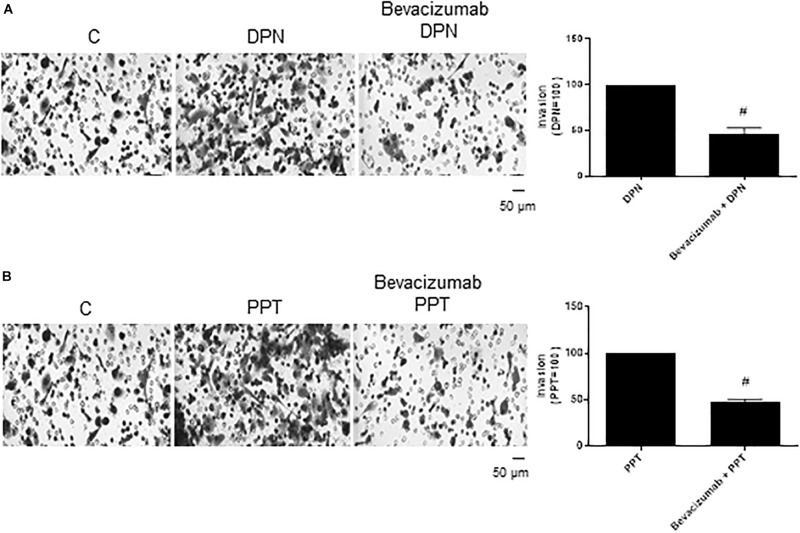
Effect of VEGF specific inhibitor Bevacizumab on DPN or PPT-induced PC-3 cells invasion. Cells in serum free culture medium were seeded in Thincert^®^ chambers with polyethylene terephthalate membranes pre-coated with phenol red-free Matrigel. These chambers were placed in 24-well plates containing culture medium with 10% FBS in the lower chamber. Cells in upper chambers were incubated in the absence (C, control) and presence of ERβ-selective agonist DPN (10 nM) **(A)** or ERα-selective agonist PPT (10 nM) **(B)** for 48 h at 37°C. Cells were also untreated or pretreated with VEGF specific inhibitor Bevacizumab (25 ng) for 30 min. Incubation was continued in the absence and presence of DPN or PPT for 48 h at 37°C **(A,B)**. The membranes containing the invaded cells (under the surface of membrane), were photographed. Images of three random microscope fields, in duplicate, were captured using an inverted optical microscope. The areas of invaded cells were determined by Image J software. Results were plotted (mean ± SEM) in relation to control (*C* = 100) or in relation to agonists subtracted from the control (agonists = 100). #Significantly different from DPN or PPT (*P* < 0.05, Student *t*-test). Scale bar as indicated. Images are representative of three different experiments (the control and all treatments were performed in the same experiment; thus, the same control was used for **A,B**).

The complex β-catenin/TCF/LEF may activate the transcription of the vascular endothelial growth factor (VEGF) in different cells (36–38), thereby stimulating angiogenesis and tumor growth. To explore the role of VEGF on PC-3 cells invasion, these cells were treated with VEGF inhibitor Bevacizumab. Bevacizumab decreased the effect of both agonists DPN (54%) and PPT (53%) on invasion of PC-3 cells (Figures 7A,B), suggesting the involvement of the VEGF in this process induced by both estrogen receptors.

The treatment with Bevacizumab alone did not have any effect on invasion of PC-3 cells (Supplementary Figure S2).

**FIGURE 8:**
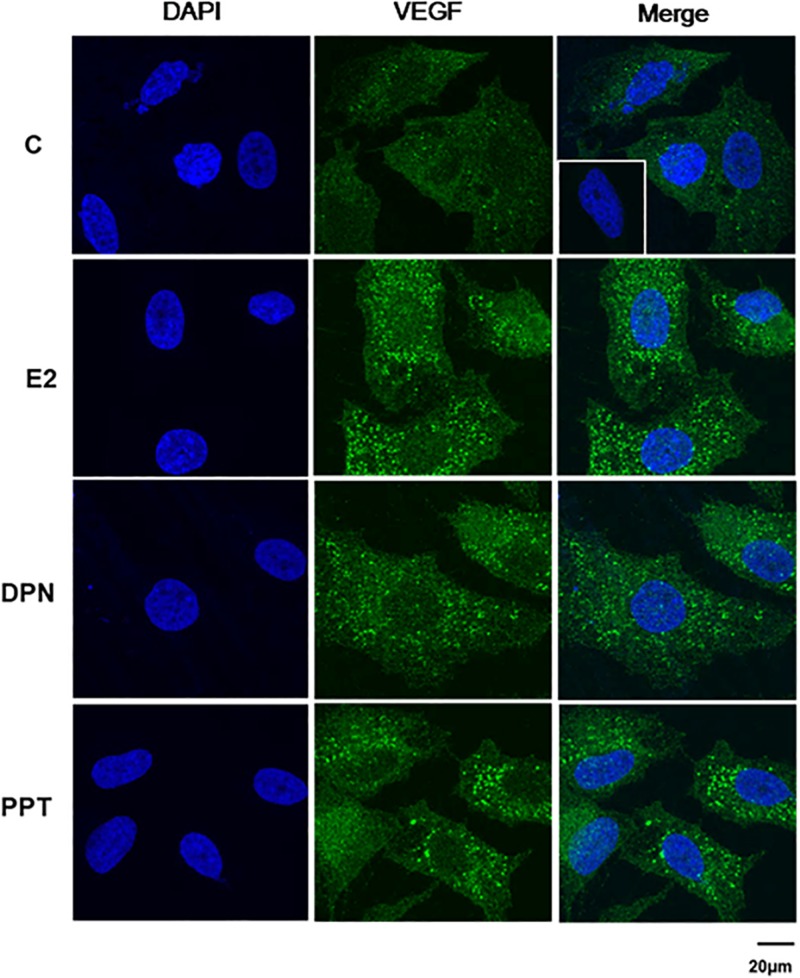
Effects of 17β-estradiol (E2), ERβ-selective agonist DPN, and ERα-selective agonist PPT on VEGFA expression in PC-3 cells. Cells were incubated in the absence (C, control) and presence of E2 (10 nM), DPN (10 nM), or PPT (10 nM) for 48 h at 37°C. Immunostaining (green) was detected using a rabbit polyclonal antibody raised against a peptide 1–140 of VEGFA of human origin and Alexa Fluor 488-labeled secondary antibody. Nuclei were stained with DAPI (blue). Negative control was performed using normal rabbit serum at the same dilution of the antibody (insert). Scale bar as indicated. The data shown are representative of two different experiments performed in duplicate.

VEGFA immunostaining was found mainly in the cytoplasm near the nucleus in control (C) and treated-cells (Figure 8). E2, DPN, and PPT (10 nM, 48 h) increased the expression of VEGFA compared to control (C’) (Figure 8). The upregulation of VEGFA induced by E2 was inhibited by pretreatment with PKF 118–310 (Supplementary Figure S3). Similar results were observed with DPN and PPT (Supplementary Figure S3). PKF 118–310 alone did not have any effect on VEGF expression (Supplementary Figure S3). All together, these results indicate that ER/β-catenin/TCF-LEF is involved on VEGFA expression.

## Discussion

Estrogen receptors ERα and ERβ are expressed in both human post pubertal prostate epithelial cell (PNT1A) and androgen-independent prostate cancer cell lines (PC-3, derived from bone metastasis, and DU-145, derived from brain metastasis) (20, 21). A greater expression of ERα (unpublished data) and ERβ (21) was detected in DU-145 cells compared to PC-3 cells, suggesting that, in the regulation of the expression of these receptors, distinct androgen-independent mechanism is involved.

Many investigations about the expression and role of ERα and ERβ in prostate cancer have been performed and conflicting results described [reviewed by (4, 5)]. We now report that the activation of ERβ promotes the increase of migration, invasion and anchorage-independent growth of PC-3 cells. Furthermore, the activation of ERα also plays a role in invasion and anchorage-independent growth of PC-3 cells.

Activation of ERβ by ERβ-selective agonists (DPN and ERB-041) increased the migration of PC-3 cells. On the other hand, ERα-selective agonist PPT did not modify cell migration. Consistent with these results, study has shown that the activation of ERβ by DPN also promotes survival and migration of CPEC cell line, established from prostate cancer patients (19). Furthermore, the expression of ERβ5 (ERβ splice variant) in PC-3 cells increased the cell migration, and the expression of ERβ2 and ERβ5 increased the invasion, but did not change the proliferation of the cells (39). It is important to emphasize that ERβ splice variants do not bind ligands[reviewed by (40)], but dimers may be observed with ERβ (ERβ1). In the present study, it was also shown that ERβ is involved in the invasion of PC-3 cells. All together, these results support an oncogenic role for ERβ.

Androgen receptors activate multiple extranuclear signaling pathways involved in prostate cancer progression, including WNT/β-catenin pathway [reviewed by (41)]. In the nucleus, β-catenin requires interaction with TCF/LEF transcription factors and other co-activators, such as CBP or p300, and activates the transcription of the target genes involved in cell proliferation, migration and invasion of different cancer cells [reviewed by (41–43)], including prostate cancer (25, 32, 44). After androgen-deprivation, the interaction of β-catenin with TCF4 is enhanced and partly involved with androgen-independent growth of prostate cancer cells [(45); reviewed by (46)]. Furthermore, crosstalk between activation of ERβ and β-catenin expression was shown in PC-3 cells and the involvement of β-catenin on cell proliferation (18).

The compound that disrupts the complex β-catenin-TCF/LEF, PKF 118–310, inhibited proliferation (18), migration, invasion, size and number of the colony formed by PC-3 cells induced by DPN (present study). All together, these results indicate the involvement of ERβ/β-catenin in all cellular characteristics of tumor development *in vitro*. It is important to mention that in the proximal β-catenin promoter gene was detected consensus regions for Sp-1 (specific protein transcription factor), c-Fos, c-Jun, and GATA-3 (zinc finger transcription factor) (18). They are potential elements that may be involved in the transcriptional regulation of β-catenin gene directly by ERs. Consistent with these findings, in human colon and breast cancer cells, ERα and β-catenin precipitate within the same immunocomplexes, reciprocally enhances the transactivation of cognate reporter genes (47). Further experiments are needed to explore the direct or indirect transcriptional regulation of β-catenin gene by ERs in PC-3 cells. The signaling pathway PI3K/AKT is also involved in the upregulation of β-catenin induced by activation of ERβ in PC-3 cells (18).

In PC-3 cells, the activation of ERα by ERα-selective agonist PPT for 24 h did not modify the expression of β-catenin (unpublished data), Cyclin D2, cell number in the S phase (DNA synthesis) of cell cycle (18) and cell migration (present study). On the other hand, the activation of ERα by PPT for 48 h increased the expression of β-catenin (unpublished data), cell invasion (27%) and the number and the size of colony formed by PC-3 cells (present study). In the invasion of PC-3 cells induced by PPT, PKF 118–310 partially reduced (59%) this effect, suggesting the involvement of ERα/β-catenin/TCF/LEF and the other signaling pathways on invasion. While, on the number and size of colony formed by PC-3 cells, PKF 118–310 completely blocked the effect of PPT, suggesting only the involvement of ERα/β-catenin/TCF/LEF in this process. Thus, ERα and ERβ may play overlapping roles on invasion and colony formed by PC-3 cells.

Cellular responses activated by ERα or ERβ might depend on a plethora of factors [reviewed by (4, 5, 8, 11, 48)], including the crosstalk between ERα and ERβ [(49), (20), (21)], the ratio of the expression of both ERs and their intracellular distribution (21), the presence of endogenous inhibitors and the availability of transcriptional co-regulator, the ligand concentration and time of ligand incubation, that differentially activate ERα or ERβ and/or their mediated responses in target cells [reviewed in (4, 11, 50)]. It appears that additional studies are needed to depict a clear picture of the role of ERα and ERβ in prostate cancer.

The complex β-catenin/TCF/LEF activates the transcription of vascular endothelial growth factor (VEGF) and other target genes in different cells (36–38), thereby stimulating angiogenesis and tumor growth. VEGF is overexpressed in prostate cancer cells (51), and may play a role in the androgen-dependent to castration-resistant disease [(52, 53); reviewed by (54, 55)].

In PC-3 cells, expression, regulation and role of VEGF were explored. Transcriptional and post-transcriptional regulations of VEGF signaling have been shown in prostate cancer [reviewed by (54)]. Four TCF/LEF-binding sites were identified within the 5′-promoter region of human VEGF gene (56). VEGF promoter is also regulated by other transcription factors, such as, hypoxia-inducible factors (HIFs), specific protein-1 (Sp-1), multiple members of the nuclear receptor family [reviewed by (54)], including ER/ERE (estrogen response elements) as previously showed by Hyder et al. (57). Indeed, E2, DPN and PPT induced an increase of VEGFA expression in PC-3 cells. This upregulation of VEGFA was inhibited by pretreatment with PKF 118–310, suggesting the involvement of ERs/β-catenin/TCF/LEF in the regulation of VEGFA expression. A better understanding of the mechanisms transcriptional and post-transcriptional, involved in the regulation of VEGFA by ERs in PC-3 cells, needs to be explored.

VEGF inhibitor Bevacizumab (Avastin) decreased the effect of both selective agonists DPN (54%) and PPT (53%) on invasion of PC-3 cells, indicating the involvement of VEGF and also the others factors on invasion of these cells. Bevacizumab binds with high affinity to all isoforms of human VEGF and neutralizes its biological activity through a steric blocking of the VEGF binding to its receptors FLT-1 (VEGFR-1) and KDR (VEGFR-2) [reviewed by (58)]. All together, these results suggest that ERs/β-catenin/TCF/LEF and VEGF are involved on PC-3 cells invasion. It is important to emphasize that other intracellular signaling pathways may be involved in β-catenin/TCF/LEF activation and prostate cancer invasion (23). The role of VEGF/VEGFR alone and together with estrogen/ERs remains to be explored in prostate cancer.

Studies have shown mis-regulation of multiple pathways in CRPC, reflecting the heterogeneity of the tumors and combined therapy targeting more than one pathway can be effective.

## Conclusion

In conclusion, this study provides novel insights into the signatures and molecular mechanisms of ERβ in androgen-independent prostate cancer cells PC-3. In PC-3 cells, the activation of ERβ increases the expression and signaling of β-catenin and induces migration, cell invasion and colony formation. The activation of ERα (48 h) also enhances β-catenin signaling and cell invasion and colony formation. Furthermore, ERs/β-catenin/TCF/LEF and VEGF are involved on PC-3 cells invasion.

## Data Availability Statement

All datasets generated for this study are included in the article/Supplementary Material.

## Ethics Statement

All experimental procedures were approved by the Research Ethical Committee at Escola Paulista de Medicina-Universidade Federal de São Paulo (#4330100615).

## Author Contributions

AL and CV performed the experiments, organized the database, performed the statistical analysis, and wrote the first draft of the manuscript. CP revised the data and literature about the topic. All authors contributed to the concept and design of the study, manuscript revision, and read and approved the submitted version.

## Conflict of Interest

The authors declare that the research was conducted in the absence of any commercial or financial relationships that could be construed as a potential conflict of interest.

## Supplementary Material

FIGURE S1 Effects of ERβ-selective antagonist PHTPP and compound that disrupts the complex β-catenin-TCF/LEF PKF 118–310 on PC-3 cells migration. Cells were incubated in culture medium containing mitomycin C (10 μg/L) to avoid cell proliferation. Cells were wounded and then incubated in the absence (C, control, basal level of cellular function) and presence of ERβ-selective antagonist PHTPP (10 nM) or compound that disrupts the complex β-catenin-TCF/LEF transcription factor PKF 118–310 (100 nM) for 24 h at 37°C. Photographs of the same area of the wound were taken at 0 and 24 h. Images were captured using an inverted optical microscope and analyzed by Micrometrics SE Premium 4 Software. The areas that were occupied by migrating cells after 24 h of incubation (control and treated cells) were calculated by subtracting the background levels at 0 h. Results were plotted (mean ± SEM) in relation to control (*C* = 1). No statistical difference was observed from control (C) (*P* > 0.05, Student *t*-test). Images are representative of three to four different experiments.

FIGURE S2 Effects of ERβ-selective antagonist PHTPP, ERα-selective antagonist MPP, compound that disrupts the complex β-catenin-TCF/LEF PKF 118–310 and VEGF specific inhibitor Bevacizumab on PC-3 cells invasion. Cells in serum free culture medium were seeded in Thincert^®^ chambers with polyethylene terephthalate membranes pre-coated with phenol red-free Matrigel. These chambers were placed in 24-well plates containing culture medium with 10% FBS in the lower chamber. Cells in upper chambers were incubated in the absence (C, control basal level of cellular function) and presence of ERβ-selective antagonist PHTPP (10 nM), ERα-selective antagonist MPP (10 nM), compound that disrupts the complex β-catenin-TCF/LEF transcription factor, PKF 118–310 (100 nM) or VEGF specific inhibitor Bevacizumab (25 ng) for 48 h at 37°C. The membranes containing the invaded cells (under the surface of membrane), were photographed. Images of three random microscope fields, in duplicate, were captured using an inverted optical microscope. The areas of invaded cells were determined by Image J software. Results were plotted (mean ± SEM) in relation to control (*C* = 100). No statistical difference was observed from control (C) (*P* > 0.05, Student *t*-test). Scale bar as indicated. Images are representative of three different experiments.

FIGURE S3 Effects of the compound that disrupts the complex β-catenin-TCF/LEF PKF 118–310 on E2-, DPN- or PPT-induced VEGFA expression in PC-3 cells. Cells were incubated in the absence (C, control) and presence of E2 (10 nM), DPN (10 nM), or PPT (10 nM) for 48 h at 37°C. Cells were also untreated or pretreated with a compound that disrupts the complex β-catenin-TCF/LEF transcription factor PKF 118–310 (100 nM) for 30 min. Incubation was continued in the absence and presence of E2, DPN, or PPT for 48 h at 37°C. Immunostaining (green) was detected using a rabbit polyclonal antibody raised against a peptide 1–140 of VEGFA of human origin and Alexa Fluor 488-labeled secondary antibody. Nuclei were stained with DAPI (blue). Negative control was performed using normal rabbit serum at the same dilution of the antibody (insert). Scale bar as indicated. The data shown are representative of two different experiments performed in duplicate.
