# Supplementary figures and images for: Estrogen Receptors Promote Migration, Invasion and Colony Formation of the Androgen-Independent Prostate Cancer Cells PC-3 Through β-Catenin Pathway
Source: Front Endocrinol (Lausanne). 2020 Apr 9;11:184. doi: 10.3389/fendo.2020.00184 (PMC7160699; doi:10.3389/fendo.2020.00184)

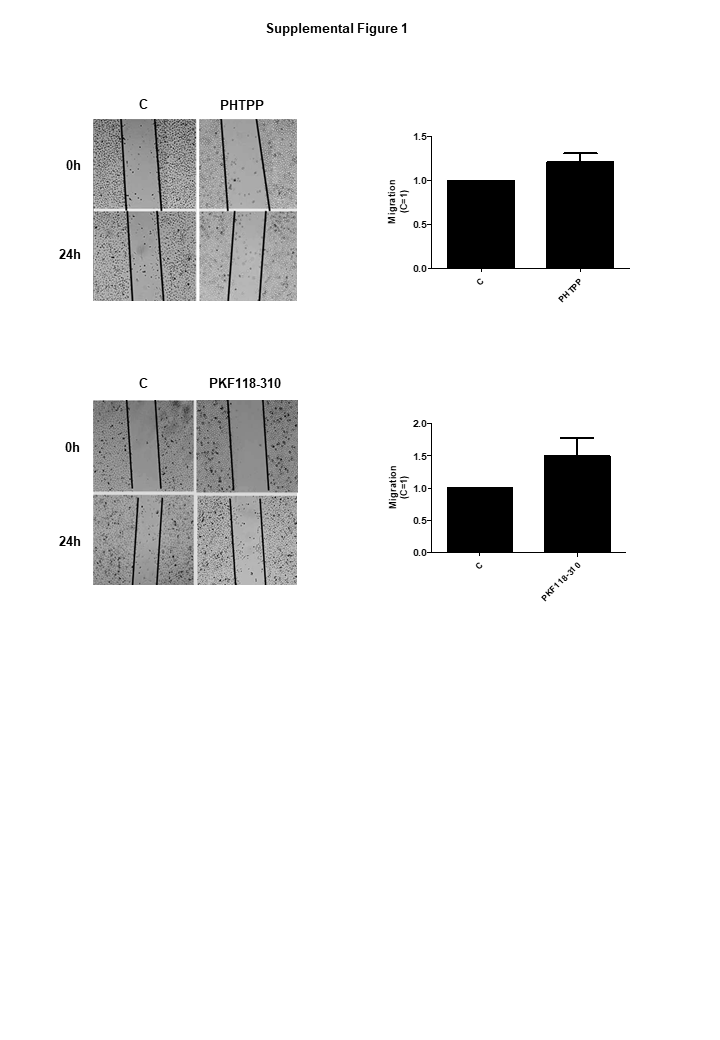

Supplement: FIGURE S1 — Effects of ERβ-selective antagonist PHTPP and compound that disrupts the complex β-catenin-TCF/LEF PKF 118–310 on PC-3 cells migration. Cells were incubated in culture medium containing mitomycin C (10 μg/L) to avoid cell proliferation. Cells were wounded and then incubated in the absence (C, control, basal level of cellular function) and presence of ERβ-selective antagonist PHTPP (10 nM) or compound that disrupts the complex β-catenin-TCF/LEF transcription factor PKF 118–310 (100 nM) for 24 h at 37°C. Photographs of the same area of the wound were taken at 0 and 24 h. Images were captured using an inverted optical microscope and analyzed by Micrometrics SE Premium 4 Software. The areas that were occupied by migrating cells after 24 h of incubation (control and treated cells) were calculated by subtracting the background levels at 0 h. Results were plotted (mean ± SEM) in relation to control (C = 1). No statistical difference was observed from control (C) (P > 0.05, Student t-test). Images are representative of three to four different experiments. [file Image_1.TIF]

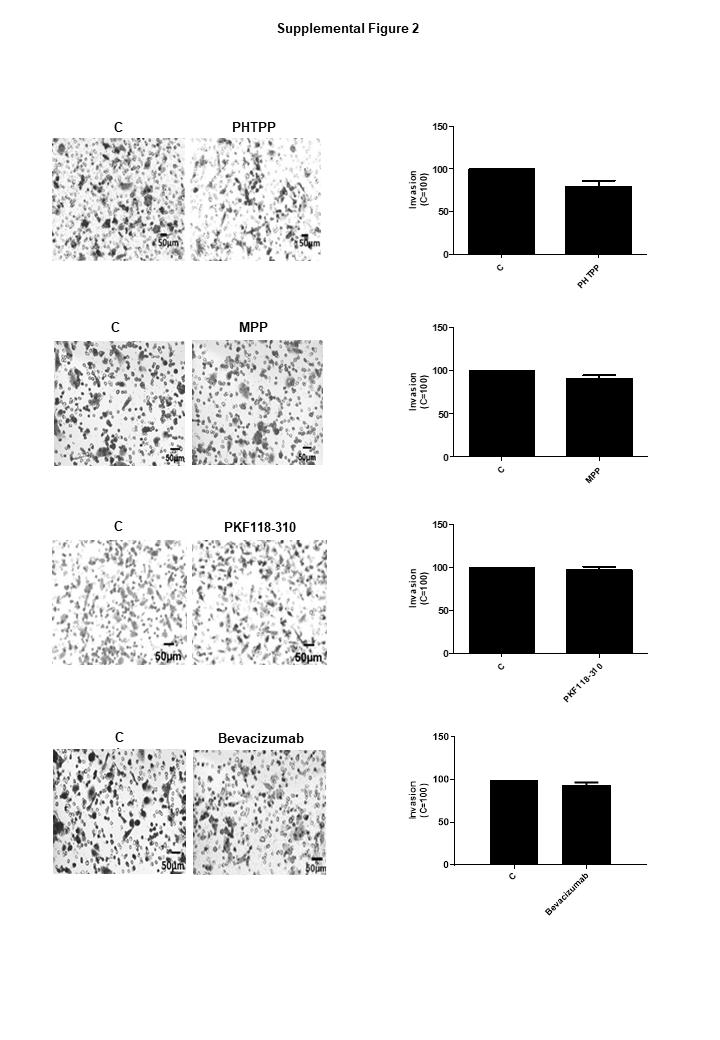

Supplement: FIGURE S2 — Effects of ERβ-selective antagonist PHTPP, ERα-selective antagonist MPP, compound that disrupts the complex β-catenin-TCF/LEF PKF 118–310 and VEGF specific inhibitor Bevacizumab on PC-3 cells invasion. Cells in serum free culture medium were seeded in Thincert® chambers with polyethylene terephthalate membranes pre-coated with phenol red-free Matrigel. These chambers were placed in 24-well plates containing culture medium with 10% FBS in the lower chamber. Cells in upper chambers were incubated in the absence (C, control basal level of cellular function) and presence of ERβ-selective antagonist PHTPP (10 nM), ERα-selective antagonist MPP (10 nM), compound that disrupts the complex β-catenin-TCF/LEF transcription factor, PKF 118–310 (100 nM) or VEGF specific inhibitor Bevacizumab (25 ng) for 48 h at 37°C. The membranes containing the invaded cells (under the surface of membrane), were photographed. Images of three random microscope fields, in duplicate, were captured using an inverted optical microscope. The areas of invaded cells were determined by Image J software. Results were plotted (mean ± SEM) in relation to control (C = 100). No statistical difference was observed from control (C) (P > 0.05, Student t-test). Scale bar as indicated. Images are representative of three different experiments. [file Image_2.TIF]

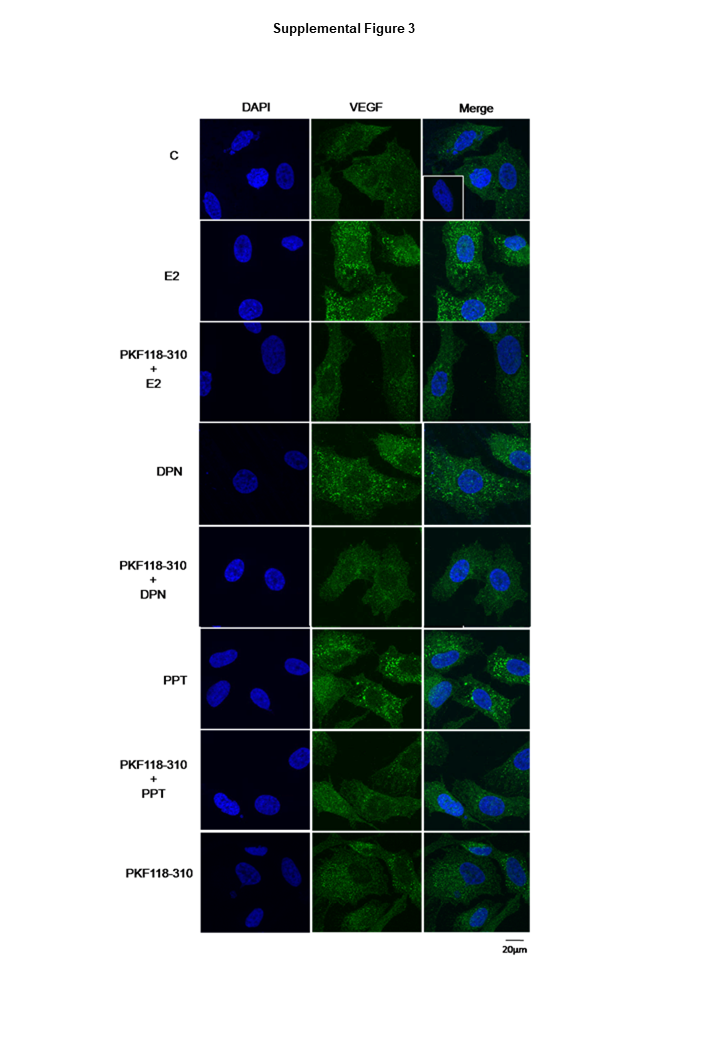

Supplement: FIGURE S3 — Effects of the compound that disrupts the complex β-catenin-TCF/LEF PKF 118–310 on E2-, DPN- or PPT-induced VEGFA expression in PC-3 cells. Cells were incubated in the absence (C, control) and presence of E2 (10 nM), DPN (10 nM), or PPT (10 nM) for 48 h at 37°C. Cells were also untreated or pretreated with a compound that disrupts the complex β-catenin-TCF/LEF transcription factor PKF 118–310 (100 nM) for 30 min. Incubation was continued in the absence and presence of E2, DPN, or PPT for 48 h at 37°C. Immunostaining (green) was detected using a rabbit polyclonal antibody raised against a peptide 1–140 of VEGFA of human origin and Alexa Fluor 488-labeled secondary antibody. Nuclei were stained with DAPI (blue). Negative control was performed using normal rabbit serum at the same dilution of the antibody (insert). Scale bar as indicated. The data shown are representative of two different experiments performed in duplicate. [file Image_3.TIF]
